# Supplementary material for: EnzML: multi-label prediction of enzyme classes using InterPro signatures
Source: BMC Bioinformatics. 2012 Apr 25;13:61. doi: 10.1186/1471-2105-13-61 (PMC3483700; doi:10.1186/1471-2105-13-61)
Supplement: Addtional file 5 — The Java code to format the data files, evaluate and predict. The file enzml_java_code.tar.gz contains the Java code used to format database data to ARFF and XML formats, to execute cross and train-test (jackknife) evaluations and to record evaluation results to database. More information is included in the readme.txt file and the Javadoc files. The code can be used with a MySQL database. To use a different database software, other JDBC drivers might be required. [file 1471-2105-13-61-S5.gz › java_code/enzml2011/doc/uk/ac/ed/inf/enzml/weka/package-summary.html]

uk.ac.ed.inf.enzml.weka


---


|  |  |  |
| --- | --- | --- |
| **Overview** \| **Package** \| Class \| **Use** \| **Tree** \| **Deprecated** \| **Index** \| **Help** | |  |
| **PREV PACKAGE**   NEXT PACKAGE | **FRAMES**    **NO FRAMES**     **All Classes** |  |


---

## Package uk.ac.ed.inf.enzml.weka

| **Class Summary** | |
| --- | --- |
| **Arff** | Creates an ARFF file: checks file properties, loads data from database, fills and writes to file the ARFF attributes, classes and instances, generates auxiliary files if needed. |
| **ArffProperties** | Contains the properties for a Weka ARFF file It contains: 1. |
| **ArffPropsTable** | Class to keep the name and column types for the database table to store the ARFF file parameters (the data files for Weka or Mulan machine learning) |
| **ArffPropsTableManager** | Writes into a database table the pointers needed to build an arff file (the name of the arff file, the query to select instances, attributes, class values etc) TODO integrate arff recording to database with mulan experiment recording |
| **ArffPropsTableReader** | Reads properties from the table where the arff records are collected. |
| **AttributeFactory** | The attribute factory produces binary and nominal Weka attributes. |
| **DataSetChecker** | Checks that all data (or file paths) needed to build the ARFF file is available |
| **DataSetDbLoader** | Loads data (instances, attribute values, class values) from database using the queries in the data Properties. |
| **DataSetGenerator** | Generates and fills the Weka instances. |
| **DataSetManager** | DataSet generates a collection of Weka instances (rows of the ARFF file), their attributes (column names) and their attribute values (cell values) The last attribute is the class attribute. |
| **DataSetWriter** | Class |
| **IdentifiedSparseInstance** | Class |
| **InstancesFiller** | Generates and fills individual instances for a weka data set. |

---


|  |  |  |
| --- | --- | --- |
| **Overview** \| **Package** \| Class \| **Use** \| **Tree** \| **Deprecated** \| **Index** \| **Help** | |  |
| **PREV PACKAGE**   NEXT PACKAGE | **FRAMES**    **NO FRAMES**     **All Classes** |  |


---
